# Circulating high sensitivity C reactive protein concentrations and risk of lung cancer: nested case-control study within Lung Cancer Cohort Consortium

**DOI:** 10.1136/bmj.k4981

**Published:** 2019-01-03

**Authors:** David C Muller, Tricia L Larose, Allison Hodge, Florence Guida, Arnulf Langhammer, Kjell Grankvist, Klaus Meyer, Qiuyin Cai, Alan A Arslan, Anne Zeleniuch-Jacquotte, Demetrius Albanes, Graham G Giles, Howard D Sesso, I-Min Lee, J Michael Gaziano, Jian-Min Yuan, Judith Hoffman Bolton, Julie E Buring, Kala Visvanathan, Loic Le Marchand, Mark P Purdue, Neil E Caporaso, Øivind Midttun, Per M Ueland, Ross L Prentice, Stephanie J Weinstein, Victoria L Stevens, Wei Zheng, William J Blot, Xiao-Ou Shu, Xuehong Zhang, Yong-Bing Xiang, Woon-Puay Koh, Kristian Hveem, Cynthia A Thomson, Mary Pettinger, Gunnar Engström, Hans Brunnström, Roger L Milne, Meir J Stampfer, Jiali Han, Mikael Johansson, Paul Brennan, Gianluca Severi, Mattias Johansson

**Affiliations:** 1 Genetic Epidemiology Group, International Agency for Research on Cancer, Lyon, France; 2 Department of Epidemiology and Biostatistics, Imperial College London, Norfolk Place, London W2 1PG, UK; 3 KG Jebsen Center for Genetic Epidemiology, Department of Public Health and Nursing, Faculty of Medicine and Health Sciences, Norwegian University of Science and Technology, Trondheim, Norway; 4 Cancer Epidemiology and Intelligence Division, Cancer Council Victoria, Melbourne, VIC, Australia; 5 Centre for Epidemiology and Biostatistics, Melbourne School of Population and Global Health, The University of Melbourne, Parkville, VIC, Australia; 6 HUNT Research Centre, Department of Public Health and Nursing, Norwegian University of Science and Technology, Levanger, Norway; 7 Department of Medical Biosciences, Umeå University, Umeå, Sweden; 8 Bevital AS, Bergen, Norway; 9 Division of Epidemiology, Department of Medicine, Vanderbilt Epidemiology Center and Vanderbilt-Ingram Cancer, Vanderbilt University School of Medicine, Nashville, TN, USA; 10 Department of Obstetrics and Gynecology, New York University School of Medicine, New York, NY, USA; 11 Department of Population Health and Environmental Medicine, New York University School of Medicine, New York, NY, USA; 12 Division of Cancer Epidemiology and Genetics, National Cancer Institute, National Institutes of Health, Bethesda, MD, USA; 13 Department of Epidemiology, Harvard TH Chan School of Public Health, Boston, MA, USA; 14 Division of Aging, Department of Medicine, Brigham and Women’s Hospital, Boston, MA, USA; 15 Division of Preventive Medicine, Department of Medicine, Brigham and Women’s Hospital, Boston, MA, USA; 16 Boston VA Medical Center, Boston, MA, USA; 17 UPMC Hillman Cancer Center, University of Pittsburgh, USA; 18 Department of Epidemiology, Graduate School of Public Health, University of Pittsburgh, USA; 19 George W Comstock Center for Public Health Research and Prevention Health Monitoring Unit, Department of Epidemiology, Johns Hopkins University Bloomberg School of Public Health, Baltimore, MD, USA; 20 Epidemiology Program, Cancer Research Center of Hawaii, University of Hawaii, Honolulu, HI, USA; 21 Division of Cancer Epidemiology and Genetics, National Cancer Institute, NIH, Bethesda, MD, USA; 22 Department of Clinical Science, University of Bergen, Bergen, Norway; 23 Laboratory of Clinical Biochemistry, Haukeland University Hospital, Bergen, Norway; 24 Division of Public Health Sciences Fred Hutchinson Cancer Research Center, Seattle, WA, USA; 25 Epidemiology Research Program, American Cancer Society, Atlanta, GA, USA; 26 Channing Division of Network Medicine, Department of Medicine, Brigham and Women’s Hospital and Harvard Medical School, Boston, MA, USA; 27 State Key Laboratory of Oncogene and Related Genes and Department of Epidemiology, Shanghai Cancer Institute, Renji Hospital, Shanghai Jiaotong University School of Medicine, Shanghai, China; 28 Duke-NUS Medical School Singapore, Singapore; 29 Health Promotion Sciences, Mel and Enid Zuckerman College of Public Health, University of Arizona, Tucson, AZ, USA; 30 Department of Clinical Science in Malmö, Lund University, Malmö, Sweden; 31 Pathology, Department of Clinical Sciences Lund, Laboratory Medicine Region Skåne, Lund University, Lund, Sweden; 32 Department of Nutrition, Harvard TH Chan School of Public Health, Boston, MA, USA; 33 Department of Epidemiology, Richard M Fairbanks School of Public Health, Indiana University, Indianapolis, IN, USA; 34 Melvin and Bren Simon Cancer Center, Indiana University, Indianapolis, IN, USA; 35 Department of Radiation Sciences, Umeå University, Umeå, Sweden; 36 Italian Institute for Genomic Medicine (IIGM), Torino, Italy; 37 Centre de Recherche en Epidemiologie et Santé des Populations (CESP) UMR1018 Inserm, Facultés de Médicine Université Paris-Saclay, UPS, UVSQ, Villejuif, France

## Abstract

**Objectives:**

To conduct a comprehensive analysis of prospectively measured circulating high sensitivity C reactive protein (hsCRP) concentration and risk of lung cancer overall, by smoking status (never, former, and current smokers), and histological sub-type.

**Design:**

Nested case-control study.

**Setting:**

20 population based cohort studies in Asia, Europe, Australia, and the United States.

**Participants:**

5299 patients with incident lung cancer, with individually incidence density matched controls.

**Exposure:**

Circulating hsCRP concentrations in prediagnostic serum or plasma samples.

**Main outcome measure:**

Incident lung cancer diagnosis.

**Results:**

A positive association between circulating hsCRP concentration and the risk of lung cancer for current (odds ratio associated with a doubling in hsCRP concentration 1.09, 95% confidence interval 1.05 to 1.13) and former smokers (1.09, 1.04 to 1.14) was observed, but not for never smokers (P<0.01 for interaction). This association was strong and consistent across all histological subtypes, except for adenocarcinoma, which was not strongly associated with hsCRP concentration regardless of smoking status (odds ratio for adenocarcinoma overall 0.97, 95% confidence interval 0.94 to 1.01). The association between circulating hsCRP concentration and the risk of lung cancer was strongest in the first two years of follow-up for former and current smokers. Including hsCRP concentration in a risk model, in addition to smoking based variables, did not improve risk discrimination overall, but slightly improved discrimination for cancers diagnosed in the first two years of follow-up.

**Conclusions:**

Former and current smokers with higher circulating hsCRP concentrations had a higher risk of lung cancer overall. Circulating hsCRP concentration was not associated with the risk of lung adenocarcinoma. Circulating hsCRP concentration could be a prediagnostic marker of lung cancer rather than a causal risk factor.

## Introduction

Lung cancer is the leading cause of mortality related to cancer,^1^ accounting for 1.7 million deaths worldwide every year.^2^ Tobacco smoke exposure is known to cause most lung cancer cases,^1^ but a deeper understanding of intermediate factors that influence lung cancer pathogenesis is limited. In general, inflammation has been proposed to be an important risk factor for cancer, but the extent to which inflammation could drive the risk of lung cancer is unclear.^3^

C reactive protein (CRP) is an acute phase inflammatory protein that is synthesised in the liver in response to low grade inflammation.^4^ ^5^ High sensitivity CRP (hsCRP) responds as a sensitive but non-specific biomarker for systemic inflammation. Two independent case-control studies nested in the Prostate, Lung, Colorectal, and Ovarian Cancer Screening Trial showed a positive association between circulating CRP concentrations and risk of lung cancer for current and former smokers.^6^ More recently, an inverse association between CRP and the risk of lung cancer was reported for female never smokers, an important subgroup that the Prostate, Lung, Colorectal, and Ovarian Cancer Screening Trial was not adequately powered to assess.^7^

Both the low cost and increased availability of point-of-care CRP tests in the primary care setting warrant further investigation into the potential use of CRP as a risk biomarker for lung cancer development.^8^ ^9^ ^10^ The primary objective of our study was to comprehensively investigate the relation between circulating hsCRP concentration and the risk of lung cancer for never, former, and current smokers. The secondary objective of our study was to conduct a risk discrimination analysis to evaluate whether circulating hsCRP concentration combined with self reported smoking information could better discriminate between current smokers at low and high risk of developing lung cancer, compared with self reported smoking information alone. This study used prediagnostic serum or plasma samples from 5299 individual incidence-density matched case-control pairs in the Lung Cancer Cohort Consortium, which included 20 prospective cohorts from Asia, Europe, Australia, and the United States.

## Methods

### Study population

All prospective cohorts with frozen baseline plasma or serum samples that were members of the US National Cancer Institute Cohort Consortium in 2009 were invited to participate in the Lung Cancer Cohort Consortium. A total of 20 prospective cohorts from Asia, Europe, Australia, and the US were invited and agreed to participate in the Lung Cancer Cohort Consortium. At recruitment, research participants provided written informed consent to their respective cohorts, and the Lung Cancer Cohort Consortium project was approved by the institutional review boards of each participating institution. Participants in each cohort were followed up for incident cancer diagnoses and vital status, predominantly by linkage to population registers. Further details about Lung Cancer Cohort Consortium cohort recruitment and participant follow-up procedures have been published,^11^ and are available in the supplementary materials.

### Selection of cases and controls

We defined lung cancer cases as all invasive cancers with ICD-O-2 (international classification of diseases for oncology, 2nd edition) codes C34.0 to C34.9. Former and never smokers were intentionally oversampled to improve statistical power in analyses stratified by smoking. We randomly selected controls for each case from risk-sets of patients who were alive and free of cancer at the time of diagnosis of their index case (incidence density matching). Cases and controls were individually matched by cohort, sex (male or female), age (initially ±1 year, relaxed to ±3 years), ethnic group (US cohorts only), date of blood draw (initially ±1 month, relaxed to ±3 months), and number of freeze-thaw cycles of their blood sample (0 or 1). We further matched cases and controls by smoking status in five categories: never smokers, short and long term quitters among former smokers (<10 years, ≥10 years since quitting), and light and heavy smokers among current smokers (<15 cigarettes per day, ≥15 cigarettes per day). Our self reported smoking covariates (smoking status, smoking duration, and smoking intensity) include tobacco use from cigarettes, cigars, and pipes. By convention, we refer to smoking intensity as cigarettes per day, even though the cigarettes per day variable in the current analysis also includes information on cigars and pipes. After matching and biochemical analyses, we used a total of 5299 case-control pairs in the current analysis, including 2496 current smoker pairs, 1498 former smoker pairs, and 1305 never smoker pairs.

### Biochemical analysis

Centralised biochemical analyses of serum or plasma hsCRP and serum or plasma cotinine were performed at the BEVITAL Laboratory in Bergen, Norway. Quantification of serum or plasma hsCRP was conducted by using Immuno-MALDI-MS.^12^ For hsCRP, the lower limit of detection was 0.1 μg/mL, the within-day coefficient of variation was 3% to 6%, and the between-day coefficient of variation was 3% to 7%. Quantification of serum or plasma cotinine was performed by liquid chromatography-mass spectrometry (LC-MS/MS).^13^ For cotinine, the lower limit of detection was 1 nmol/L, the within-day coefficient of variation was 2% to 3%, and the between-day coefficient of variation was 6%. The intraclass correlation coefficient of cotinine was 0.89-0.95.^14^

### Statistical analyses

We used conditional logistic regression to calculate odds ratios and 95% confidence intervals for incident lung cancer per fourths of hsCRP concentration. We also analysed hsCRP concentration as a continuous exposure variable by using the base 2 logarithm of hsCRP (OR_log2CRP_). OR_log2CRP_ estimates can be interpreted as the relative risk associated with a doubling in hsCRP concentration. Estimates from conditional logistic regression models were conditioned on matched case set and adjusted for fourths of circulating cotinine concentration. P values were from likelihood ratio tests of the hsCRP terms.

In addition to controlling for tobacco exposure by the smoking-matched study design, we further adjusted models for circulating cotinine concentration (in fourths). All risk analyses were conducted overall, and stratified by smoking status (never, former, and current, to have sufficient sample size in each group for the stratified estimates), and region (Asia, Europe and Australia (combined to ensure sufficient sample size), and the US). Further stratified risk analyses were conducted by sociodemographic and clinical variables, including the following: sex, age at baseline (years), body mass index (kg/m^2^), and time from blood draw to diagnosis (years). We also stratified by histological subtype (large cell, small cell, squamous cell, adenocarcinoma, other, and unknown or missing). Heterogeneity of these stratified estimates was evaluated using the likelihood ratio test of the interaction terms between hsCRP and each covariate.

To evaluate if circulating hsCRP concentration combined with self reported smoking information (CRP model) could improve discrimination between current smokers at high and low risk of lung cancer compared with self reported smoking information alone (base model), we calculated the area under the receiver operating characteristic curve (AUC) for each model. Self reported smoking models included smoking status; number of cigarettes, cigars, and pipes per day; and duration of smoking (number of years participant regularly smoked cigarettes, pipes, or cigars). Both cigars and pipes per day and smoking duration were modelled using restricted cubic splines with 3 degrees of freedom. These models were fitted to data from current smokers. We further fitted these models restricting the analysis to cases diagnosed within the first two years of follow-up and their matched controls (time from blood draw to diagnosis of two years or less).

All statistical analyses were conducted using R version 3.4.2.^15^

### Patient and public involvement

No patients were involved in setting the research question or the outcome measures, nor were they involved in developing plans for recruitment, design, or implementation of the study. No patients were asked to advise on interpretation or writing up of results.

## Results

Table 1 shows that our study sample included 5299 incident lung cancer cases and 5299 individually matched controls. Overall, slightly more participants were male (54%). Participants from cohorts in Asia and Europe and Australia were also predominantly male (69% and 58%, respectively). Current smokers accounted for nearly half of the overall study sample (47%, 2496 case-control pairs), with former and never smokers contributing approximately one quarter each (former 28%, 1498 case-control pairs; never 25%, 1305 case-control pairs). Median age at cohort recruitment was 60 years (table 1). Cases and controls had similar characteristics, on average, except for body mass index, for which a smaller proportion of cases had body mass index greater than 25 kg/m^2^. Circulating cotinine showed considerable variability among self reported current smokers, whereas most never and former smokers did not have detectable cotinine concentrations (supplementary materials, fig 1).

**Table 1:** Distribution of participant characteristics, overall and by region. Values are numbers (percentages)

Characteristic		Overall			Asia			Europe and Australia			US	
		Control	Case		Control	Case		Control	Case		Control	Case
Total		5299 (100)	5299 (100)		1757 (100)	1757 (100)		1159 (100)	1159 (100)		2383 (100)	2383 (100)
Sex:												
Men		2873 (54)	2873 (54)		1218 (69)	1218 (69)		671 (58)	671 (58)		984 (41)	984 (41)
Women		2426 (46)	2426 (46)		539 (31)	539 (31)		488 (42)	488 (42)		1399 (59)	1399 (59)
Smoking status:												
Never		1305 (25)	1305 (25)		592 (34)	592 (34)		147 (13)	147 (13)		566 (24)	566 (24)
Former		1498 (28)	1498 (28)		175 (10)	175 (10)		325 (28)	325 (28)		998 (42)	998 (42)
Current		2496 (47)	2496 (47)		990 (56)	990 (56)		687 (59)	687 (59)		819 (34)	819 (34)
Age (years) at baseline:												
17-54		1539 (29)	1519 (29)		432 (25)	428 (24)		331 (29)	334 (29)		776 (33)	757 (32)
55-59		997 (19)	991 (19)		377 (21)	365 (21)		251 (22)	251 (22)		369 (15)	375 (16)
60-64		1238 (23)	1256 (24)		436 (25)	464 (26)		336 (29)	327 (28)		466 (20)	465 (20)
65-86		1525 (29)	1533 (29)		512 (29)	500 (28)		241 (21)	247 (21)		772 (32)	786 (33)
Education:												
Less than high school		1670 (32)	1773 (33)		877 (50)	893 (51)		578 (50)	643 (55)		215 (9)	237 (10)
Completed high school		774 (15)	753 (14)		228 (13)	242 (14)		175 (15)	155 (13)		371 (16)	356 (15)
Vocational school		904 (17)	878 (17)		274 (16)	285 (16)		198 (17)	177 (15)		432 (18)	416 (17)
Some college		707 (13)	670 (13)		192 (11)	168 (10)		127 (11)	105 (9)		388 (16)	397 (17)
College graduate		492 (9)	518 (10)		113 (6)	102 (6)		63 (5)	61 (5)		316 (13)	355 (15)
Graduate studies		707 (13)	640 (12)		64 (4)	59 (3)		8 (1)	10 (1)		635 (27)	571 (24)
Missing		45 (1)	67 (1)		9 (1)	8 (0)		10 (1)	8 (1)		26 (1)	51 (2)
Body mass index (kg/m^2^):												
14.1-24.9		2769 (52)	2973 (56)		1291 (73)	1347 (77)		434 (37)	519 (45)		1044 (44)	1107 (46)
25.0-29.9		1795 (34)	1658 (31)		420 (24)	364 (21)		524 (45)	457 (39)		851 (36)	837 (35)
30.0-59.5		673 (13)	604 (11)		46 (3)	46 (3)		199 (17)	182 (16)		428 (18)	376 (16)
Missing		62 (1)	64 (1)		0 (0)	0 (0)		2 (0)	1 (0)		60 (3)	63 (3)
hsCRP (µg/mL):												
0.00388-0.731		1325 (25)	1180 (22)		610 (35)	540 (31)		275 (24)	243 (21)		440 (18)	397 (17)
0.732-1.76		1320 (25)	1242 (23)		468 (27)	455 (26)		312 (27)	280 (24)		540 (23)	507 (21)
1.77-4.13		1326 (25)	1384 (26)		372 (21)	390 (22)		304 (26)	314 (27)		650 (27)	680 (29)
4.14-87.9		1328 (25)	1493 (28)		307 (17)	372 (21)		268 (23)	322 (28)		753 (32)	799 (34)

Table 2 shows that the median time between blood draw and lung cancer diagnosis for cases was 6.8 years. Table 2 shows that most lung cancer cases, overall and by region, were diagnosed with adenocarcinoma, followed by squamous cell, small cell, and large cell carcinomas. Overall, 38% of cancers in the sample were adenocarcinomas (43% of those cancers diagnosed in the US, compared with 35% for Asia and 34% for Europe and Australia). 

**Table 2:** Clinical characteristics of lung cancer cases, overall and by region. Values are numbers (percentages)

Characteristics	Overall	Asia	Europe and Australia	US
Time (years) from blood draw to diagnosis:				
0.0-1.9	583 (11)	257 (15)	82 (7)	244 (10)
2.0-4.9	1325 (25)	463 (26)	134 (12)	728 (31)
5.0-9.9	1631 (31)	613 (35)	362 (31)	656 (28)
10.0-35.6	1577 (30)	424 (24)	581 (50)	572 (24)
Histology:				
Large cell	173 (3)	16 (1)	46 (4)	111 (5)
Small cell	484 (9)	98 (6)	146 (13)	240 (10)
Squamous cell	831 (16)	319 (18)	226 (19)	286 (12)
Adenocarcinoma	2030 (38)	608 (35)	398 (34)	1024 (43)
Other	595 (11)	124 (7)	178 (15)	293 (12)
Unknown or missing	1186 (22)	592 (34)	165 (14)	429 (18)

**Fig 1:**
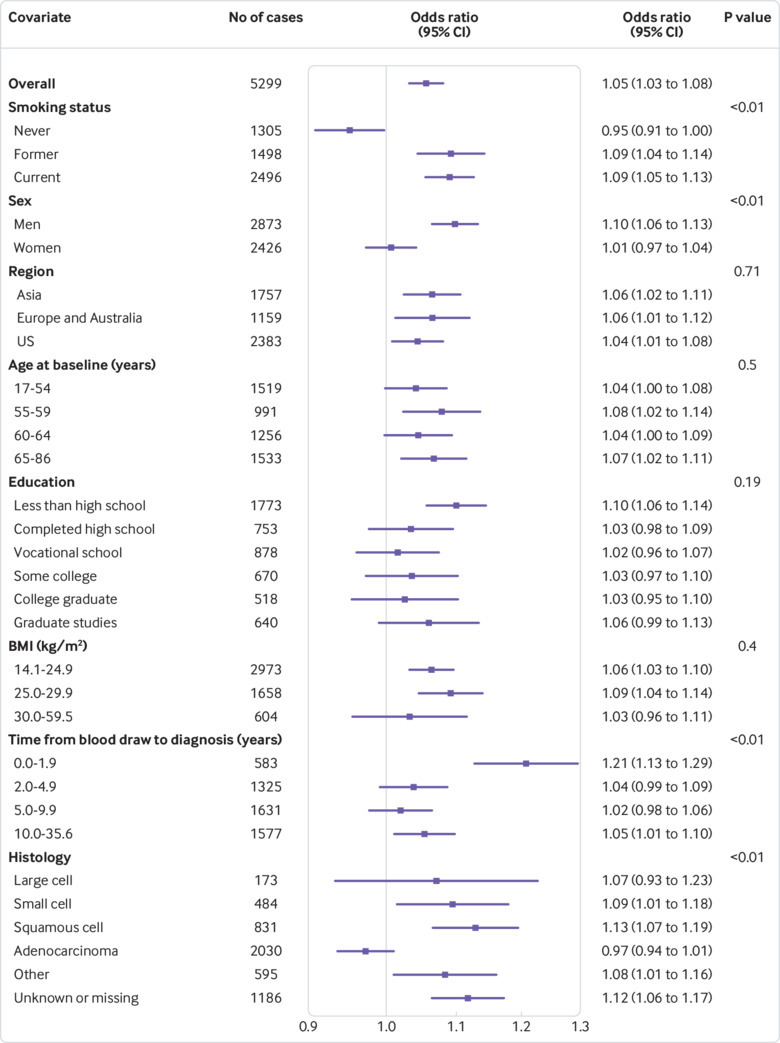
Odds ratios for doubling in high sensitivity C reactive protein (hsCRP) concentration, overall and by participant characteristics. P values are from likelihood ratio tests of the interaction between hsCRP and each covariate

Table 3 shows that higher hsCRP concentration was positively associated with the overall risk of lung cancer (odds ratio 1.05, 95% confidence interval 1.03 to 1.08, P< 0.001 for trend). Associations between hsCRP concentration and the risk of lung cancer were slightly stronger in models without adjustment for circulating cotinine (supplementary materials, table 1). Figure 1 shows that the association was most apparent for current (odds ratio 1.09, 95% confidence interval 1.05 to 1.13) and former smokers (1.09, 1.04 to 1.14). We did not observe a positive association for never smokers (0.95, 0.91 to 1.00; fig 1). Figure 1 shows that the positive association between hsCRP concentration and the risk of lung cancer was stronger for patients who were diagnosed in the first two years of follow-up (odds ratio 1.21, 95% confidence interval 1.13 to 1.29, P< 0.01 for interaction). There was also some indication that the association was stronger for participants in the less than high school education group, though there was little statistical evidence for heterogeneity, and this is likely driven by the greater proportion of current smokers in this group (supplementary materials, table 2).

**Table 3:** Odds ratios for lung cancer by high sensitivity C reactive protein (hsCRP) concentration in fourths, and for a doubling in hsCRP concentration

hsCRP concentration	No of controls	No of cases	Odds ratio (95% CI)	P value
Categorical (μg/mL):				
0.00388-0.731	1325	1180	1.00 (ref)	<0.001
0.732-1.76	1320	1242	1.05 (0.93 to 1.17)	
1.77-4.13	1326	1384	1.17 (1.04 to 1.31)	
4.14-87.9	1328	1493	1.26 (1.12 to 1.41)	
Continuous:				
Doubling in concentration	5299	5299	1.05 (1.03 to 1.08)	<0.001

**Fig 2:**
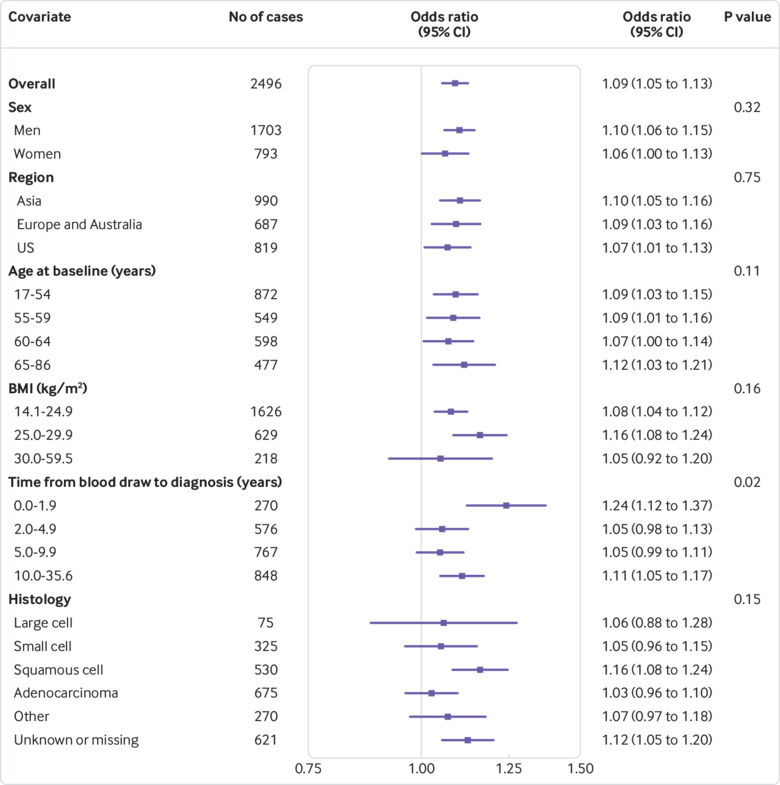
Odds ratios for doubling in high sensitivity C reactive protein (hsCRP) concentration for current smokers by participant characteristics. P values are from likelihood ratio tests of the interaction between hsCRP and each covariate

**Fig 3:**
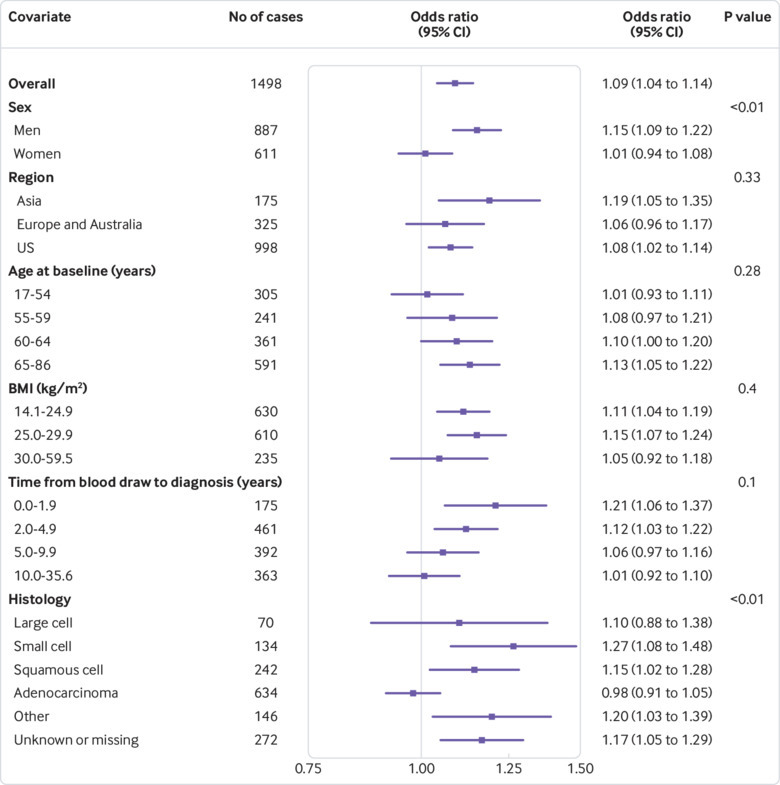
Odds ratios for doubling in high sensitivity C reactive protein (hsCRP) concentration for former smokers by participant characteristics. P values are from likelihood ratio tests of the interaction between hsCRP and each covariate

Further stratified risk analyses showed that the association between hsCRP concentration and the risk of lung cancer differed by histological subtype (P<0.01 for interaction for overall risk of lung cancer, fig 1; P=0.15 for interaction for current smokers, fig 2; and P<0.01 for former smokers, fig 3). For instance, although we observed a strong and consistent association between hsCRP concentration and the risk of squamous cell lung cancer among current (odds ratio 1.16, 95% confidence interval 1.08 to 1.24, fig 2) and former (1.15, 1.02 to 1.28, fig 3) smokers, no corresponding association was observed for adenocarcinoma among current (1.03, 0.96 to 1.10, fig 2) and former (0.98, 0.91 to 1.05, fig 3) smokers.

**Fig 4:**
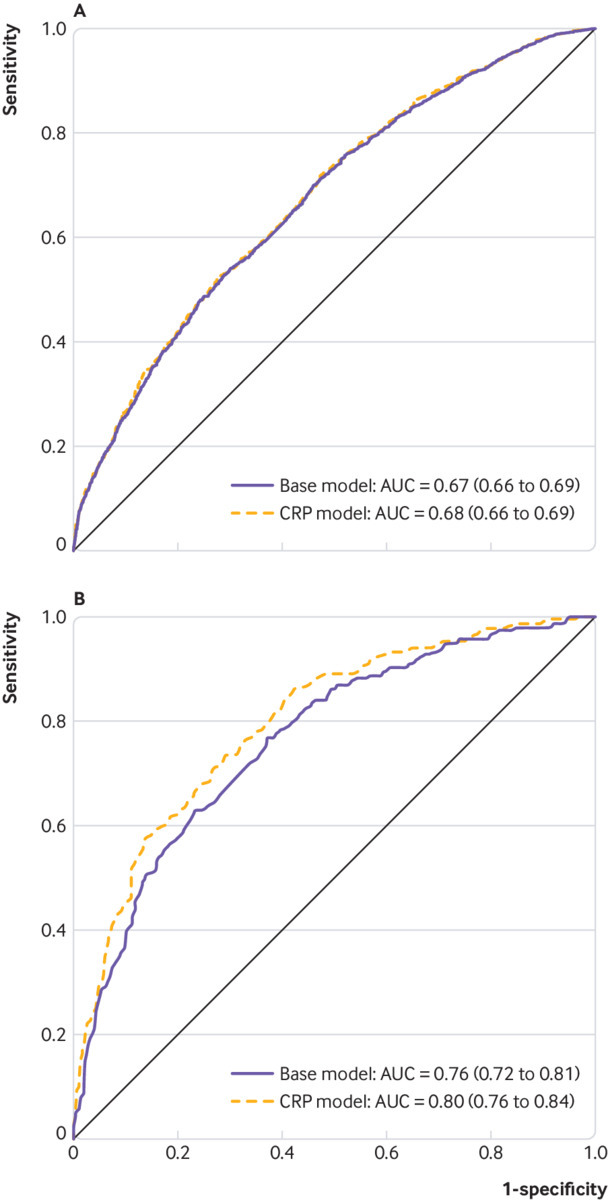
Current smokers **A** overall (n=2199 case-control pairs) and **B** with time from blood draw to diagnosis ≤2 years (n=224 case-control pairs)

Figure 4A shows that for current smokers overall, combining hsCRP concentration and self reported smoking information into an integrated model (CRP model) did not improve discriminative performance when compared with the base model, which only included self reported smoking variables (AUC_CRP model_ 0.68, 95% confidence interval 0.66 to 0.69; AUC_Base model_ 0.67, 95% confidence interval 0.66 to 0.69). Figure 4B shows that for current smokers with a time from blood draw to diagnosis of two years or less, the CRP model did provide further risk discriminative information (AUC_CRP model_ 0.80, 95% confidence interval 0.76 to 0.84) compared with self reported smoking alone (AUC_Base model_ 0.76, 95% confidence interval 0.72 to 0.81).

## Discussion

We conducted a comprehensive analysis of the relation between circulating hsCRP concentration and the risk of lung cancer by using prediagnostic samples from 20 prospective cohorts in the Lung Cancer Cohort Consortium. Former and current smokers with higher hsCRP concentrations had an increased risk for some histological subtypes of lung cancer, but not for adenocarcinoma. We observed a substantial association between hsCRP concentration and the risk of lung cancer in the first two years of follow-up.

### Interpretation and implications

Unresolved chronic inflammation can generate reactive oxygen species and reactive nitrogen species that promote tumour growth through angiogenesis and cell proliferation.^16^ Given our finding of a weak and inconsistent association between hsCRP concentration and the risk of lung cancer in the longer term (>2 years after blood draw), systemic inflammation does not seem to be a likely driver of early stage lung cancer, or hsCRP concentration might not be capturing the risk, or both. In turn, the etiological role of inflammation in the development of lung carcinogenesis remains debated.^17^ ^18^

As chronic inflammation could arise owing to an immune response to tobacco smoke exposure,^19^ and considering the lack of association we observed for never smokers, residual confounding by smoking exposure could explain some of the associations we observed for the period beyond two years from blood draw. This interpretation would also be in line with our observation of higher hsCRP concentration being associated with histological subtypes most strongly associated with tobacco smoking, in contrast with the lack of association we observed for adenocarcinoma. The histological types that are strongly driven by tobacco smoke exposure will be more susceptible to residual confounding by smoking than adenocarcinoma, for which the risk increase by smoking is smaller.^20^

In stratified analysis by time from blood draw, we observed a stronger positive association between hsCRP concentration and the risk of lung cancer in the first two years of follow-up, particularly for current smokers. This result suggests that higher hsCRP concentrations could be indicative of the presence of preclinical disease state, as opposed to being a causal risk factor for lung cancer. If hsCRP concentration is indicative of a preclinical disease state, it is natural to consider it as an addition to smoking-based risk factors for screening eligibility. Our risk discrimination analysis—in which we found no improvement in discrimination overall and a small improvement for diagnoses in the first two years of follow-up—suggests that hsCRP concentration alone is unlikely to improve selection of patients for lung cancer screening. Further, given the lack of association between hsCRP concentration and the risk of lung adenocarcinoma, it would not be appropriate to use clinical CRP tests to rule out the presence of lung cancer during diagnostic work-up of patients who are symptomatic.

### Strengths and weaknesses

This study has several strengths, including the use of prediagnostic assays of hsCRP from a large and diverse study population of individually matched case-control pairs within 20 prospective cohorts from several regions around the world. The large study sample allowed for robust risk analyses across all smoking status categories, and we were further able to adjust for circulating cotinine concentrations—an objective measure of recent tobacco exposure. Never and former smokers were oversampled allowing for well powered smoking-stratified risk analyses. Our 1305 never smoker case-control pairs make this the largest prospective risk biomarker study for lung cancer in never smokers. These patients who were never smokers represent an increasingly important subgroup that previous studies were underpowered to evaluate.^6^ ^21^ ^22^

Our study was limited by the use of hsCRP measurements from one time point for each patient. Individual repeated samples would have been particularly useful for better evaluation of circulating hsCRP concentrations in the years leading up to a lung cancer diagnosis.

### Conclusions

Former and current smokers with higher hsCRP concentrations had a greater risk of some histological subtypes of lung cancer, but not adenocarcinoma. We observed a stronger association between higher hsCRP concentration and the risk of lung cancer in the first two years of follow-up, indicating that circulating hsCRP concentrations might reflect a prediagnostic disease state as opposed to a causal risk for lung cancer.

**What is already known on this topic**

Previous studies have shown that C reactive protein (CRP), a marker of systemic inflammation, is associated with the risk of lung cancer
The studies have not been sufficiently large to provide precise estimates of association by smoking status (never, former, or current smokers)

**What this study adds**

High sensitivity CRP (hsCRP) concentration is associated with the risk of lung cancer in former and current smokers, but not in never smokers
The association is strongest in the first two years after blood draw
hsCRP concentration is not associated with the risk of lung adenocarcinoma

## Web Extra

Extra material supplied by the author

**Supplementary materials**: Supplementary materials, tables 1 and 2, and figure 1

**Supplementary materials**: Women’s Health Initiative investigators

## Notes

Contributors: DCM and TLL contributed jointly to the study. MaJ****and PB organised the Lung Cancer Cohort Consortium study. DCM, TLL, and FG conducted the data analysis. Data was interpreted by all authors. TLL, DCM and MaJ wrote the first draft of the manuscript. All authors read and approved the final manuscript. DCM and MaJ had full access to the data in the study and can take responsibility for the integrity of the data and the accuracy of the data analysis. DCM and MaJ are guarantors of the article. The corresponding author attests that all listed authors meet authorship criteria and that no others meeting the criteria have been omitted.

Ethical approval: The protocol of the Lung Cancer Cohort Consortium was approved by the institutional review board of the International Agency for Research on Cancer. The recruitment, data collection, and follow-up of the participating cohorts was approved by local institutional review boards.

Data Sharing: No additional data are available.

The study guarantors (DCM and MaJ) affirm that this manuscript is an honest, accurate, and transparent account of the study being reported; that no important aspects of the study have been omitted; and that any discrepancies from the study as planned have been explained.
