# Supplementary material for: Circulating high sensitivity C reactive protein concentrations and risk of lung cancer: nested case-control study within Lung Cancer Cohort Consortium
Source: BMJ. 2019 Jan 3;364:k4981. doi: 10.1136/bmj.k4981 (PMC6315896; doi:10.1136/bmj.k4981)
Supplement: Supplementary file 1 — Supplementary materials: Supplementary materials, tables 1 and 2, and figure 1 [file muld045971.ww1.pdf]

Supplementary Materials for "Circulating hs-CRP concentrations and lung cancer risk: a nested case-control study within the Lung Cancer Cohort Consortium (LC3)"

Supplementary Methods

Study population

We invited all prospective cohort studies that in 2009 were members in the US National Cancer Institute (NCI) Cohort Consortium to participate in the study. Additional inclusion criteria included the occurrence of at least 200 incident lung cancer cases with baseline questionnaire data and either plasma or serum samples available. Twenty-one cohorts fulfilled those criteria and accepted to participate, but one cohort failed to send biospecimens and were not included, leaving the 20 cohorts described below.

Brief description of the participating cohorts in the Lung Cancer Cohort Consortium

1) **US cohorts**

<u>The Women's Health Initiative (WHI)</u>

WHI is a long-term health study of 161,808 post-menopausal women aged 50 to 79 years at 40 clinical centers throughout the U.S. WHI comprises a Clinical Trial (CT) component (68,132 women), and an Observational Study (OS) component (93,676 women), and has included several extension studies. Some detailed descriptions of WHI have been previously presented[1 2].The CT evaluated two forms of postmenopausal hormone therapy, a low-fat dietary pattern intervention, and calcium and vitamin D supplementation in a randomized, controlled fashion, in a partial factorial design. The hormone therapy component findings led to major reductions in the use of hormone therapy worldwide, and are thought to have led to noteworthy reductions in breast cancer incidence.

In the present study lung cancer cases occurring during the follow-up of WHI cohorts since enrolment (1993-1998) among non-smoking women, were matched 1-1 to corresponding non-smoking lung cancer free controls, for serum and DNA analyte comparisons.

<u>The Southern Community Cohort Study (SCCS)</u>

The Southern Community Cohort Study (SCCS)[3] is a prospective cohort of African and non-African Americans which during 2002-2009 enrolled approximately 86,000 residents aged 40-79 years across 12 southern states. Recruitment occurred mainly at community health centers, institutions providing basic health services primarily to the medically uninsured, so that the cohort includes many adults of lower income and educational status. Each study participant completed a detailed baseline questionnaire, and nearly 90% provided a biologic specimen (approximately 45% a blood sample and 45% buccal cells). Follow-up of the cohort is conducted by linkage to national mortality registers and to state cancer registries.

<u>Prostate, Lung, Colorectal and Ovarian Cancer Screening Trial (PLCO)</u>

The PLCO study, a randomized trial aimed at evaluating the efficacy of screening in reducing cancer mortality, recruited approximately 155,000 men and women age 55 to 74 years from 1992 to 2001[4]. Screening for lung cancer among participants in the intervention arm included a chest x-ray at baseline followed by either three annual x-rays (for current or former smokers at enrollment) or two annual x-rays (for never smokers); participants in the control arm received routine health care. Screening-arm participants provided data on sociodemographic factors, smoking behavior, anthropometric characteristics, medical history, and family history of cancer, as well as blood samples annually for the first 6 years of the study (baseline [T0] and T1 through T5). Lung cancers were ascertained through annual questionnaires mailed to the participants, and positive reports were followed up by abstracting medical records or death certificates. Follow-up in the trial as of July 2009 was 96.7%.

We conducted a nested case-control study within the screening arm of the PLCO trial. As of December 31, 2004, 898 lung cancers were diagnosed among the 77,464 participants. Patients were excluded because of missing baseline questionnaire, previous history of any cancer, diagnosis of multiple cancers during follow-up, missing smoking information at baseline, missing consent for utilization of biologic specimens for etiologic studies, or unavailability/insufficient quantity of serum or DNA specimens. Hemolyzed vials were excluded. Participants for this nested case-control study were sampled from the intervention arm.

Controls were individuals free of cancer at the time of a case's lung cancer diagnosis. Controls were individually matched to lung cancer patients on sex, date of birth +/- 1 year with a possible relaxation to 5 years, race, study year of blood draw, date of blood draw +/- 1 month (with a possible relaxation to 3/6 months), time of blood draw (6AM-9AM, 9AM-12PM, other), smoking categories (smoking status at enrollment, never, former, or current smoker; cumulative amount of smoking (0 to 29, 30 to 39, 40 to 49, and 50+ pack-years), with additional matching for time since quitting (< 15 years and ≥ 15 years) for former smokers, cigarettes per day smoked, and number of days in the study.

The New York University Women's Health Study (NYUWHS)

The New York University Women's Health Study (NYUWHS) is a prospective cohort study of women enrolled at a mammography screening center in New York City. From March 1985 through June 1991, 14,274 women between the ages of 34 and 65 were enrolled in the study. Because the original focus of the study was endogenous hormones and breast cancer, women who had taken hormone medications in the 6 months preceding baseline enrolment were not eligible for the study.

At the time of enrolment, data on demographics, anthropometric measures, medical history, reproductive and lifestyle variables were collected through self-administered questionnaires after written informed consent was obtained. Incident lung cancer cases were identified through active follow-up of the cohort conducted with questionnaires mailed approximately every two to four years and record linkages with state tumor registries in New York, New Jersey, and Florida, as well as record linkage with the National Death Index (NDI). Medical records were obtained to verify reported cancer outcomes.

The American Cancer Society Cancer Prevention Study-II (CPS-II) Nutrition Cohort

The CPS-II Nutrition Cohort is a prospective study of cancer incidence and mortality among 86,404 men and 97,786 women. The CPS-II Nutrition Cohort, which is described in detail elsewhere[5], was initiated in 1992 as a subgroup of CPS-II, a prospective study of cancer mortality involving approximately 1.2 million Americans begun in 1982. Participants in the CPS-II Nutrition Cohort were recruited from CPS-II members who resided in 21 states and were between the ages of 50 and 74 years. At enrollment in 1992/1993, participants completed a self-administered questionnaire that included demographic, medical, dietary, and lifestyle information. Follow-up questionnaires were sent to all living Nutrition Cohort members in 1997, and every two years after this to update exposure information and to ascertain newly diagnosed cancers. Between June 1998 and June 2001, blood samples were collected from a subset of CPS-II Nutrition Cohort participants (21,965 women and 17,411 men).

Incident lung cancer cases were identified through self-report on a follow-up questionnaire, linkage with state cancer registries, or death certificates. Self-reported cancers were verified through medical records.

The Campaign Against Cancer and Stroke (CLUE I) and the Campaign Against Cancer and Heart Disease (CLUE II).

The CLUE studies include two large cohorts of volunteers from Washington County, Maryland that were enrolled in 1974 and 1989, respectively. CLUE I was conducted in Washington County, Maryland, in the fall of 1974. Brief health histories and blood pressures were taken and 15 ml of blood was drawn from 26,147 volunteers (23,951 were residents of Washington County) at the time of enrollment. Linkage of the records from this program to those of a private census in the summer of 1975 indicated that almost a third of the adult population of the county had participated. CLUE II was an outgrowth of CLUE I conducted from May through October in 1989. As in CLUE I, a brief health history was obtained and 20 ml of blood was drawn. A blood sample was collected from 32,894 volunteers at the time of enrollment (25,076 were residents of Washington County). Participants were also given a

food frequency questionnaire to complete at home and were asked to return it with a toenail clipping of the large toe for trace metal assays. Comparisons with published figures from the 1990 Census indicted that approximately 30 percent of adult residents had participated.

The Multiethnic Cohort (MEC)

The MEC includes over 215,000 men and women aged 45-75 years at recruitment from five different racial/ethnic groups (African Americans, Japanese Americans, Native Hawaiians, Latinos and European Americans) in Hawaii and California[6]. The cohort was assembled in 1993-1996 by mailing a self-administered, 26-page questionnaire to obtain extensive information on demographics, medical and reproductive histories, medication use, family history of various cancers, physical activity and diet. Identification of incident cancer cases is by regular linkage with the Hawaii, Los Angeles County and California SEER registries. From 1995 to 2001, blood collection was conducted from incident cases with breast, prostate, or colorectal cancers, as well as a random sample of cohort participants to serve as controls in genomic nested case-control studies (participation rate 72% and 63%, respectively). In addition, from 2001 to 2006, blood was also collected prospectively, without regard for cancer diagnosis, from willing cohort participants. Approximately 67,000 gave a blood sample (participation rate 43%). All incident lung cancer cases diagnosed before 2010 with a pre-diagnostic blood sample were considered for inclusion in this study. Each case was matched to a control based on study site, sex, age, race/ethnicity, smoking status, hours of fasting, and date and time of blood draw.

Women's Health Study (WHS)

The WHS was a randomized trial of low-dose aspirin, vitamin E, and beta-carotene in the primary prevention of cardiovascular disease and cancer beginning in 1992 among 39,876 female US health professionals aged ≥45 years[7]. Information on major clinical, lifestyle, and dietary factors was collected via self-reports on baseline questionnaires. Women also provided baseline bloods. During more than two decades of follow-up, WHS participants reporting new cases of cancer on annual follow-up questionnaires were confirmed by medical record review by the WHS Endpoints Committee. Reports of cancer were confirmed on the basis of pathology or cytology reports or, rarely, strong clinical and radiologic or laboratory marker evidence when a pathology or cytology review was not conducted. Only confirmed cases of lung cancer were included in the present analyses, which were matched with eligible controls.

Physicians' Health Study (PHS)

The PHS I began in 1982 as a randomized trial of aspirin and beta-carotene for the primary prevention of heart disease and cancer among 22,071 male, Caucasian physicians initially aged 40 to 84 years[8], followed by the PHS II trial beginning in 1997 to evaluate beta-carotene, vitamin C, vitamin E, and a daily multivitamin on the prevention of cancer, CVD, and other endpoints. The PHS II included 14,641 men, with 7,641 participants from the PHS I plus 7,000 new physicians, for a total of 29,071 PHS participants[9]. A wide range of demographic, clinical, and lifestyle factors were assessed via baseline questionnaires, along with baseline bloods. PHS participants reported major clinical endpoints, including cancer, yearly in a mailed questionnaire and postcards every six months. Self-reported, incident lung cancer cases were confirmed through medical record review by the PHS Endpoints Committee in included in the present analyses.

The Nurses' Health Study (NHS)

The Nurses' Health Study (NHS)[10 11] was established in 1976, when 121,700 married female registered nurses aged 30 to 55 years residing in 11 States in the U.S. completed and returned a self-administered questionnaire. Questionnaires have been mailed to participants in both cohorts every 2 years since baseline to collect updated information on demographics, lifestyle factors, medical history, and disease outcomes. A semi-quantitative food frequency questionnaire (FFQ) was administered to obtain information on usual dietary intake over the previous year. The reproducibility and validity of the FFQs have been established[12-16]. The FFQ was first administered in 1980 in the NHS, and were repeated almost every 4 years thereafter. For each food item, the questionnaire specified a common serving size and queried respondents on average intake during the previous year; responses in 9 categories ranged from almost never to 6 or more per day. Most nutritional variables measured by these FFQs have been developed, tested, and refined by our group over the past 30 years (https://regepi.bwh.harvard.edu/health/).

The follow-up rate has been greater than 90%. The institutional review board at the Brigham and Women's Hospital approved the study. As approved by the committee, return of the questionnaires was considered to imply informed consent. Cases of lung cancer were self-reported by the participants or identified on their death certificates and were subsequently confirmed by medical records.

Health Professionnals Follow-up Study (HPFS)

The Health Professionals Follow-up Study (HPFS)[17] is an ongoing cohort study of 51,529 U.S. male professionals who were aged 40 to 75 years at baseline in 1986. Questionnaires have been mailed to participants every 2 years since baseline to collect updated information on demographics, lifestyle factors, mwedical history, and disease outcomes. The follow-up

rate has been greater than 90%. The institutional review board at the Harvard T.H. Chan School Public Health approved this study. As approved by the committee, return of the questionnaires was considered to imply informed consent. A semi-quantitative food frequency questionnaire (FFQ) was administered to obtain information on usual dietary intake over the previous year. The FFQ was first administered in 1986 and was repeated every 4 years thereafter. The reproducibility and validity of the FFQ have been estabished[14] [18]. For each food item, the questionnaire specified a common serving size and queried respondents on average intake during the previous year; responses in 9 categories ranged from almost never to 6 or more per day. Cases of lung cancer were self-reported by the participants or identified on their death certificates and were subsequently confirmed by medical records.

We would like to thank the participants and staff of the Nurses' Health Study and the Health Professionals Follow-up Study, for their valuable contributions as well as the following state cancer registries for their help: AL, AZ, AR, CA, CO, CT, DE, FL, GA, ID, IL, IN, IA, KY, LA, ME, MD, MA, MI, NE, NH, NJ, NY, NC, ND, OH, OK, OR, PA, RI, SC, TN, TX, VA, WA, WY. This work was supported by the National Institutes of Health (NIH) grants, UM1CA167552, UM1CA186107, P01CA87969, and R01CA49449. The content is solely the responsibility of the authors and does not necessarily represent the official views of the NIH.

2) European/Australian cohorts

The Melbourne Collaborative Cohort Study (MCCS)

The MCCS is a prospective cohort study of 41,514 participants (17,045 men and 24,469 women) aged 27-88 years at recruitment[19]; 99.3% of whom were aged 40-69 years. Recruitment occurred between 1990 and 1994. Southern European migrants to Australia (including 5,411 Italians and 4,525 Greeks) were over-sampled to extend the range of lifestyle exposures and to increase genetic variation.

Subjects were recruited via Electoral Rolls (registration to vote is compulsory for adults in Australia), advertisements, and community announcements in local media. Comprehensive lists of Italian and Greek surnames were used to target southern European migrants in phonebooks and electoral rolls. Passive follow-up of the cohort has been conducted by record linkage to Electoral Rolls, electronic phonebooks, the Victorian Cancer Registry and death records; as well as national cancer and death records to identify events outside of Victoria.

At recruitment participant's height and weight were measured, blood samples collected and questionnaires covering lifestyle (diet, smoking, physical activity and alcohol consumption), demographics and medical history completed.

Incident lung cancer cases under 80 years of age up to 31st December 2010 were identified through record linkage.

The Malmö Diet and Cancer Study (MDCS)

The Malmö Diet and Cancer Study (MDCS) is a population-based prospective cohort study that between 1991 and 1996 recruited men and women aged 44 to 74 years of age living in Malmö, Sweden[20]. The main goal of the MDCS is to study the impact of diet on cancer incidence and mortality. It consists of a baseline examination including dietary assessment, a self-administered questionnaire, anthropometric measurements and collection of blood samples.

The Northern Sweden Health and Disease Study Cohort (NSHDS)

The Northern Sweden Health and Disease Study (NSHDS) encompasses several prospective cohorts, the current study involving study participants from the Västerbotten Intervention Project (VIP), a sub-cohort within NSHDS[21]. VIP is an ongoing prospective cohort and intervention study intended for health promotion of the general population of the Västerbotten County in northern Sweden. VIP was initiated in 1985 and all residents in the Västerbotten County were invited to participate by attending a health check-up at 40, 50 and 60 years of age. Participants were asked to complete a self-administered questionnaire including various demographic factors such as education, smoking habits, physical activity and diet. In addition, height and weight were measured and participants were asked to donate a fasting blood sample for future research. Incident lung cancer cases were identified through linkage with the regional cancer registry.

The Alpha-Tocopherol, Beta-Carotene Cancer Prevention Study (ATBC)

The ATBC Study was a randomized, double-blind, placebo-controlled, primary cancer prevention trial testing daily supplementation with α-tocopherol (50 mg/day) or β-carotene (20 mg/day), or both[22]. Between 1985 and 1988, the study enrolled and randomized 29,133 50-69 year old male cigarette smokers from southwestern Finland. Study supplementation continued for 5-8 years (median 6.1 years) until death or trial closure (April 30, 1993). At baseline, participants completed questionnaires regarding general risk factors, medical history, smoking habits, and dietary intake. Height, weight, heart rate, and blood pressure were measured by trained nurses and fasting serum samples were collected. Lung cancer cases diagnosed during follow-up through 2009 were identified through linkage with the Finnish Cancer Registry, and 200 were individually matched to 200 controls for this analysis. For the current study, study participants were selected randomly from the different arms of the ATBC study.

The Nord-Trøndelag Health Study (HUNT)

The HUNT study is a longitudinal population based study having invited all persons aged 20-100 years living in the county of Nord-Trøndelag, Norway to three data collections, HUNT1 (1984-86), HUNT2 (1995-97) and HUNT 3 (2006-08) (http://www.ntnu.edu/hunt). Each time about 95,000 persons were invited, and respectively 88%, 69% and 54% participated. Comprehensive data on life style, health status, symptoms, diseases and anthropometrics have been collected through questionnaires, interviews and clinical examinations, and in HUNT2 and HUNT3 biological material as blood and urine additionally were collected and stored. Incident cancer cases were identifiead via linkage to the Norwegian Cancer Registry.

The HUNT Study is a collaboration between HUNT Research Centre (Faculty of Medicine and Health Sciences, NTNU, Norwegian University of Science and Technology, Nord-Trøndelag County Council, Central Norway Health Authority, and the Norwegian Institute of Public Health.

3) **Asian cohorts**

The Shanghai Men's Health Study (SMHS) and the Shanghai Women's Health Study (SWHS)

The SMHS and SWHS are population-based cohort studies conducted in eight communities of urban Shanghai. Their designs and methods have been described elsewhere[23 24].

Briefly, the SWHS recruited 74,941 women during 1997-2000 (response rate: 93%) and the SMHS recruited 61,480 men during 2002-2006 (response rate: 74%). Similar methods and questionnaires were used in both studies. At baseline in-person interviews, information on sociodemographic, diet, lifestyle, occupation and medical history was obtained; height, body weight, and waist circumference were measured. Blood samples were collected from 75% of the study participants in both studies, processed within 6 hours, and stored at -70℃ until analysis.

The SMHS and SWHS have been followed up by annual record linkage with the population-based Shanghai Cancer Registry and Shanghai Vital Statistics Registry and in-person surveys every 2-3 years. Exposure information, including dietary intake, was updated in the in-person follow-up surveys. All possible matches from the linkages are checked manually and verified by home visits. Medical charts were obtained from the initial diagnostic hospitals to verify cancer diagnosis. Death certificate data from the Shanghai Vital Statistics Unit was used to identify the primary cause of death.

The studies were approved by the Institutional Review boards of the Shanghai Cancer Institute and Vanderbilt University. Informed consent was obtained from all participants.

The Singapore Chinese Health Study (SCHS)

The design of the SCHS study has been described[25 26]. Briefly, the cohort was drawn from permanent residents or citizens of Singapore who resided in government-built housing estates (86% of the Singapore population reside in such facilities). The eligible age range for cohort enrolment was 45-74 years. We restricted study subjects to the two major dialect groups of Chinese in Singapore: the Hokkiens and the Cantonese, who originated from Fujian and Guangdong provinces in Southern China, respectively. Between April 1993 and December 1998, 63,257 subjects (approximately 85% of eligible subjects) were enrolled into the cohort study. At recruitment, each study subject was interviewed in person by a trained interviewer using a structured questionnaire that emphasized current diet assessed via a validated, 165-item food frequency questionnaire. The questionnaire also requested information on demographics, lifetime use of tobacco, incense use, current physical activity, usual sleep duration, reproductive history (women only), occupational exposure, medical history, and family history of cancer.

Beginning in April 1994, a random 3% sample of cohort participants were asked to provide blood or buccal cell (if request for blood sample was denied), and spot urine samples. Eligibility for this biospecimen subcohort was extended to all surviving cohort participants starting in January 2000. By April 2005, all surviving cohort subjects had been contacted for biospecimen donation. Approximately 60% of eligible cohort participants donated biospecimens.

The cohort has been passively followed for death and cancer occurrence through regular record linkage with the population-based Singapore Cancer Registry and the Singapore Registry of Births and Deaths. Migration out of Singapore, especially among housing estate residents, is negligible. As of latest update, only 55 individuals from this cohort were known to be lost to follow-up due to migration and other reason.

A nested case-control study of incident lung cancer cases within the Singapore Chinese Health Study was established. Briefly, 422 lung cancer cases were identified among cohort participants with available prediagnostic plasma samples as of 12/31/2011. For each case, one control subject was randomly selected from all eligible cohort members who were alive and free of cancer on the date of cancer diagnosis of the index case. The control subject was individually matched to the index case by gender, dialect group (Hokkien, Cantonese), age at enrolment (±3 years), date of baseline interview (±2 year), date of biospecimen collection (±6 months), and smoking status (current, former, and never smokers). For current smokers, cases and controls were further matched by number of cigarettes per day (<15, ≥15 cigarettes/day). For former smokers, cases and controls were further matched by years since quitting smoking (<10, ≥10 years). One plasma aliquot per subject was retrieved from the biorepository and all plasma samples were sent to the laboratory (B-vital) for measurements.

The Shanghai Cohort Study (SCS)

The SCS study is a residential cohort of 18,244 men in Shanghai, China, assembled during 1986-89 when subjects were between the ages of 45 and 64 years. Approximately 80% of eligible men participated in the study. At the time of recruitment, each cohort subject was interviewed in-person by a trained nurse interviewer using a structured questionnaire that included background information, history of tobacco and alcohol use, current diet, and medical history[27 28].

At the completion of the interview, the nurse collected a 10 ml blood and a single void urine specimen from the study participant. Blood and urine samples were kept in insulated boxes with ice (0-2°C). The serum was separated from blood specimen within 3-4 hours after collection. Two sets of serum (2 ml and 1 ml, respectively) and two sets of urine samples (10 ml each) per subject have been stored at -80°C.

The cohort has been followed for the occurrence of cancer and death through routine ascertainment of new cases from the population-based Shanghai Cancer Registry and Shanghai Vital Statistics Units. To maximize the cancer findings and minimize the loss of follow-up, we have recontacted each surviving cohort member annually. Retired nurses visit the last known address of each living cohort member and record details of the interim health history of the cohort member. As of December 31, 2014, cumulatively 612 (3.4%) original subjects were lost to follow-up (i.e., persons we have no record of death and we have been unable to locate through our annual follow-up recontacts), and 574 (3.1%) refused to our continued follow-up interview (their cancer and vital status has been continually updated through record linkage analyses) after 26 years of follow-up since the beginning of the study.

A nested case-control study of incident lung cancer cases within the Shanghai Cohort Study was established. Briefly, 516 lung cancer cases were identified among cohort participants with available serum samples as of 12/31/2006. For each case, we randomly selected one control subject from all cohort members who were free of cancer and alive at the time of cancer diagnosis of the index case. Controls were matched to the index case by age at enrolment (±2 years), date of biospecimen collection (±1 month) and neighbourhood of residence at recruitment, and smoking status (current, former and never smokers) as established previously for other studies. For former smokers, cases and controls were further matched by years since quitting smoking (<10 vs ≥10 years). One serum vial per subject was retrieved from biorepository and all serum samples were sent to the laboratory (B-vital) for measurements.

Supplementary Tables

Supplementary Table 1: Odds ratios and [95% confidence intervals] for lung cancer by high sensitivity C-reactive protein (hs-CRP) concentration in quartiles, and for a doubling in hs-CRP concentration in the Lung Cancer Cohort Consortium (LC3), without adjustment for circulating cotinine.

	Controls (n)	Cases (n)	OR	95% CI
**Categorical**				
[0.00388,0.732)	1325	1180	1.00	
[0.732,1.77)	1320	1242	1.07	[0.96, 1.20]
[1.77,4.14)	1326	1384	1.19	[1.07, 1.33]
[4.14,87.9]	1328	1493	1.26	[1.15, 1.44]
**Continuous**				
Doubling in concentration	5299	5299	1.06	[1.04, 1.08]

Estimates from conditional logistic regression models conditioned on matched case set.

Supplementary Table 2: Distribution of smoking status at recruitment by level of educational attainment.

	Smoking status					
	Never		Former		Current	
Education	n	percent	n	percent	n	percent
less than high school	645	19	680	20	2118	62
completed high school	435	28	374	24	718	47
vocational school	407	23	472	26	903	51
some college	411	30	431	31	535	39
college graduate	250	25	376	37	384	38
graduate studies	440	33	627	47	280	21
missing	22	20	36	32	54	48

Supplementary Figures

Supplementary Figure 1: Box-plot of circulating cotinine concentrations by self-reported smoking status.

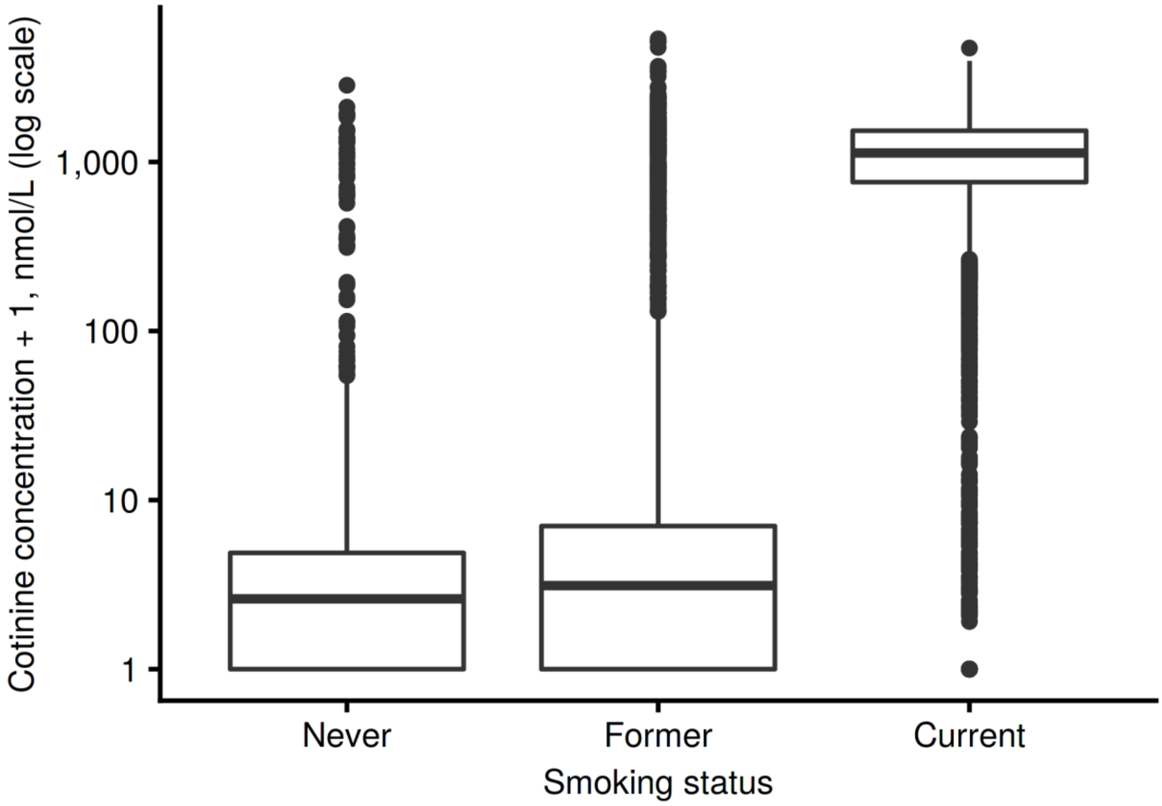

References

1. Design of the Women's Health Initiative clinical trial and observational study. The Women's Health Initiative Study Group. *Control Clin Trials* 1998;19(1):61-109.
2. Hays J, Hunt JR, Hubbell FA, Anderson GL, Limacher M, Allen C, et al. The Women's Health Initiative recruitment methods and results. *Ann Epidemiol* 2003;13(9 Suppl):S18-77.
3. Signorello LB, Hargreaves MK, Steinwandel MD, Zheng W, Cai Q, Schlundt DG, et al. Southern community cohort study: establishing a cohort to investigate health disparities. *J Natl Med Assoc* 2005;97(7):972-9.
4. Prorok PC, Andriole GL, Bresalier RS, Buys SS, Chia D, Crawford ED, et al. Design of the Prostate, Lung, Colorectal and Ovarian (PLCO) Cancer Screening Trial. *Control Clin Trials* 2000;21(6 Suppl):273S-309S.
5. Calle EE, Rodriguez C, Jacobs EJ, Almon ML, Chao A, McCullough ML, et al. The American Cancer Society Cancer Prevention Study II Nutrition Cohort: rationale, study design, and baseline characteristics. *Cancer* 2002;94(2):500-11.
6. Kolonel LN, Henderson BE, Hankin JH, Nomura AM, Wilkens LR, Pike MC, et al. A multiethnic cohort in Hawaii and Los Angeles: baseline characteristics. *Am J Epidemiol* 2000;151(4):346-57.
7. Buring JE HC. The women's health study: Rationale and background. *J Myocardial Ischemia* 1992;4:30-40.
8. Final Report on the Aspirin Component of the Ongoing Physician's Health Study. *NEJM* 1989 July;321:129-35.
9. Christen WG, Gaziano JM, Hennekens CH. Design of Physicians' Health Study II--a randomized trial of beta-carotene, vitamins E and C, and multivitamins, in prevention of cancer, cardiovascular disease, and eye disease, and review of results of completed trials. *Ann Epidemiol* 2000;10(2):125-34.
10. Belanger CF, Hennekens CH, Rosner B, Speizer FE. The nurses' health study. *Am. J. Nurs.* 1978;78(6):1039-40.
11. Colditz GA, Hankinson SE. The Nurses' Health Study: lifestyle and health among women. *Nat Rev Cancer* 2005;5(5):388-96.
12. Willett WC, Sampson L, Stampfer MJ, Rosner B, Bain C, Witschi J, et al. Reproducibility and validity of a semiquantitative food frequency questionnaire. *Am J Epidemiol* 1985;122(1):51-65.
13. Wu K, Willett WC, Fuchs CS, Colditz GA, Giovannucci EL. Calcium intake and risk of colon cancer in women and men. *J Natl Cancer Inst* 2002;94(6):437-46.
14. Rimm EB, Giovannucci EL, Stampfer MJ, Colditz GA, Litin LB, Willett WC. Reproducibility and validity of an expanded self-administered semiquantitative food frequency questionnaire among male health professionals. *Am J Epidemiol* 1992;135(10):1114-26; discussion 27-36.
15. Salvini S, Hunter DJ, Sampson L, Stampfer MJ, Colditz GA, Rosner B, et al. Food-based validation of a dietary questionnaire: the effects of week-to-week variation in food consumption. *Int. J. Epidemiol.* 1989;18(4):858-67.
16. Feskanich D, Rimm EB, Giovannucci EL, Colditz GA, Stampfer MJ, Litin LB, et al. Reproducibility and validity of food intake measurements from a semiquantitative food frequency questionnaire. *J. Am. Diet. Assoc.* 1993;93:790-96.
17. Giovannucci E, Ascherio A, Rimm EB, Colditz GA, Stampfer MJ, Willett WC. Physical activity, obesity, and risk for colon cancer and adenoma in men. *Ann Intern Med* 1995;122(5):327-34.
18. Feskanich D, Rimm EB, Giovannucci EL, Colditz GA, Stampfer MJ, Litin LB, et al. Reproducibility and validity of food intake measurements from a semiquantitative food frequency questionnaire. *J Am Diet Assoc* 1993;93(7):790-6.
19. Giles GG, English DR. The Melbourne Collaborative Cohort Study. *IARC Sci Publ* 2002;156:69-70.

20. Berglund G, Elmstahl S, Janzon L, Larsson SA. The Malmo Diet and Cancer Study. Design and feasibility. *J Intern Med* 1993;233(1):45-51.
21. Hallmans G, Agren A, Johansson G, Johansson A, Stegmayr B, Jansson JH, et al. Cardiovascular disease and diabetes in the Northern Sweden Health and Disease Study Cohort - evaluation of risk factors and their interactions. *Scand J Public Health Suppl* 2003;61:18-24.
22. The alpha-tocopherol, beta-carotene lung cancer prevention study: design, methods, participant characteristics, and compliance. The ATBC Cancer Prevention Study Group. *Ann Epidemiol* 1994;4(1):1-10.
23. Shu XO, Li H, Yang G, Gao J, Cai H, Takata Y, et al. Cohort Profile: The Shanghai Men's Health Study. *Int J Epidemiol* 2015;44(3):810-8.
24. Zheng W, Chow WH, Yang G, Jin F, Rothman N, Blair A, et al. The Shanghai Women's Health Study: rationale, study design, and baseline characteristics. *Am J Epidemiol* 2005;162(11):1123-31.
25. Hankin JH, Stram DO, Arakawa K, Park S, Low SH, Lee HP, et al. Singapore Chinese Health Study: development, validation, and calibration of the quantitative food frequency questionnaire. *Nutr Cancer* 2001;39(2):187-95.
26. Yuan JM, Stram DO, Arakawa K, Lee HP, Yu MC. Dietary cryptoxanthin and reduced risk of lung cancer: the Singapore Chinese Health Study. *Cancer epidemiology, biomarkers & prevention : a publication of the American Association for Cancer Research, cosponsored by the American Society of Preventive Oncology* 2003;12(9):890-8.
27. Ross RK, Yuan JM, Yu MC, Wogan GN, Qian GS, Tu JT, et al. Urinary aflatoxin biomarkers and risk of hepatocellular carcinoma. *Lancet* 1992;339(8799):943-6.
28. Yuan JM, Ross RK, Wang XL, Gao YT, Henderson BE, Yu MC. Morbidity and mortality in relation to cigarette smoking in Shanghai, China. A prospective male cohort study. *Jama* 1996;275(21):1646-50.
